# Inflammation, Iron, Energy Failure, and Oxidative Stress in the Pathogenesis of Multiple Sclerosis

**DOI:** 10.1155/2015/725370

**Published:** 2015-05-27

**Authors:** Lukas Haider

**Affiliations:** Department of Neuroimmunology, Center for Brain Research and Department of Biomedical Imaging and Image-Guided Therapy, Medical University of Vienna, Währinger Gürtel 18-20, 1090 Vienna, Austria

## Abstract

Multiple sclerosis is a chronic inflammatory demyelinating disease of the central nervous system. Different trigger pathologies have been suggested by the primary cytodegenerative “inside-out” and primary inflammation-driven “outside-in” hypotheses. Recent data indicate that mitochondrial injury and subsequent energy failure are key factors in the induction of demyelination and neurodegeneration. The brain weighs only a few percent of the body mass but accounts for approximately 20% of the total basal oxygen consumption of mitochondria. Oxidative stress induces mitochondrial injury in patients with multiple sclerosis and energy failure in the central nervous system of susceptible individuals. The interconnected mechanisms responsible for free radical production in patients with multiple sclerosis are as follows: (i) inflammation-induced production of free radicals by activated immune cells, (ii) liberation of iron from the myelin sheets during demyelination, and (iii) mitochondrial injury and thus energy failure-related free radical production. In the present review, the different sources of oxidative stress and their relationships to patients with multiple sclerosis considering tissue injury mechanisms and clinical aspects have been discussed.

## 1. Introduction

Multiple sclerosis is a chronic disease of the central nervous system and is associated with the formation of focal myelin loss and progressive neurodegeneration [1].

Clinically, 80% of patients with multiple sclerosis present with relapsing-remitting multiple sclerosis (RRMS), which refers to clearly defined episodes of neurological dysfunction followed by (partial) recovery. After 15–25 years, RRMS is transformed into secondary progressive multiple sclerosis (SPMS) in a majority of the patients; SPMS is characterised by progressive neurological symptoms. Patients with multiple sclerosis (10–15%) enter this neurodegenerative phase at disease onset, which is referred to as primary progressive multiple sclerosis (PPMS) [2–4].

Once patients enter the progressive phase, no currently available drug provides a significant clinical effect. Neurological decline in these patients is caused by chronic and diffuse neurodegeneration. Neurodegeneration is closely associated with inflammation, both morphologically and quantitatively, in all phases of multiple sclerosis [5, 6]. Anti-inflammatory drugs that fail to prevent disease progression are highly effective in reducing neuroinflammatory attacks in patients with RRMS. No animal models are available that mimic this enigma of the progressive phase and three nonexclusive hypotheses have been proposed as follows [7]:

- Inflammation in the relapsing-remitting and progressive phases is driven by the same mechanisms, but during progressive MS the central nervous system does not respond to currently available anti-inflammatory drugs [5], which may be caused by the closed blood-brain barrier present in progressive MS [8, 9].
- Microglia, which are under the control of intact neurons, may become chronically active due to primary neurodegeneration, axonal degeneration, and additional peripheral activation processes such as systemic inflammation [10–13].
- Multiple sclerosis may be caused by primarily cytodegenerative processes/infections, which are amplified by inflammation [14–17].

Although these models are fundamentally different, they share a common feature; that is, the tissue injury mechanisms are closely related to the production of reactive oxygen and nitrogen species.

## 2. Sources of Oxidative Stress in Multiple Sclerosis

The central role of oxidative stress has been frequently suggested in the pathogenesis of multiple sclerosis [18] based on the biochemical analysis of cerebrospinal fluid/blood samples, tissue homogenates [19–22], and animal models of multiple sclerosis [23, 24]. Oxidised DNA molecules, lipids, and protein adducts are frequently found in active multiple sclerosis lesions [24, 25] and are associated spatially and quantitatively with apoptotic oligodendrocytes and neurodegeneration in the brains of patients with multiple sclerosis [26].

## 3. Inflammation

Active inflammation and breakdown of the blood-brain barrier can be detected in the brains of patients with multiple sclerosis as gadolinium enhancing magnetic resonance imaging lesions [9, 27–29]. Although inflammatory infiltrates are present in all the stages of multiple sclerosis [5], the qualitative and quantitative composition is variable and dependent on clinical parameters (age, disease duration) and their location within the brain (meningeal, perivascular, and parenchymal). The diverse aspects of inflammation in patients with multiple sclerosis are discussed extensively [30]. Two waves of inflammatory responses can be distinguished as follows: (i) The initial/prephagocytic lesions contain few CD8 positive T-cells [17] and tissue injury is associated with the presence of activated microglia that infiltrate the parenchyma [31]; (ii) this is followed by the secondary recruitment of T-cells, B-cells, and macrophages during myelin breakdown [30].

Macrophage/microglia-derived reactive oxygen and nitrogen species trigger axonal injury [23]. The enzymes involved in the production of free radicals as well as their regulatory and catalytic subunits are upregulated in active multiple sclerosis lesion areas [32–35], where oxidative stress adducts are found most frequently [26, 36]. Experimental autoimmune encephalomyelitis (EAE) is an animal model that mimics certain phenomena observed in patients with multiple sclerosis [37]. The gene of Gp91phox encodes the catalytic component of NADPH oxidase [38]. Mice deficient in Gp91phox develop a milder form of EAE [39].

In physiological conditions, neurons, astrocytes, and oligodendrocytes express molecules that bind to receptors that are expressed on microglia. Signalling via these molecules, such as the fractalkine receptor ligand CX3CL1 [10], the membrane glycoprotein CD200 [40], the integrin associated protein myelin CD47 [41], and sialic acid alpha-2,8-linked polysialic acids [42], inhibits microglial activation [43]. Axonal degeneration can thus activate microglia that are distant from the initial site of neuronal injury due to the decreased expression of these molecules [13], which may trigger the microglia-mediated removal of debris and delivery of neurotrophic factors. Similar to other neurodegenerative diseases, such “preactivated” microglia can be more easily converted into a cytotoxic form when exposed to a proinflammatory cytokine milieu (e.g., via systemic inflammations) [44]. In addition to the randomly distributed plaques, the preactivation of microglia in EAE using Wallerian degeneration determined the location of demyelinating lesions that appeared in the ipsilateral thalamus after cortical cryoinjury and in the ipsilateral optic nerve, contralateral optic tract, and superior colliculus after unilateral eye ball enucleation [45]. Accordingly, cortical thickness in patients with multiple sclerosis is associated with the connected thalamic nucleus's neuronal cell density and the myelin content of the anatomical connection [12].

In patients with long disease durations (median > 372 months), the level of inflammatory infiltrates is similar to those found in age-matched controls [5]. Although there is a close association between oxidative stress and inflammation in active lesions and patients with RRMS, it is also pronounced in progressive MS [26, 46], which raises the question whether additional reactive oxygen and nitrogen species are present in patients with progressive MS.

## 4. Iron

In the human brain, iron is primarily stored by ferritin [47] in the myelin sheets [48, 49]. It accumulates physiologically with age, reaching a plateau at 40–50 years, depending on the anatomical structure that is analysed [50]. Experimental spinal cord injury revealed that iron can amplify oxidative damage in lesions of the central nervous system [51]. Myelin breakdown and subsequent phagocytosis of myelin debris occur at active multiple sclerosis lesions [17, 30]. It has been suggested that iron liberated into the extracellular space during the course of myelin breakdown amplifies the first wave of oxidative stress in multiple sclerosis lesions [52]. Finally, iron is taken up by macrophages and microglia. However, these cells degenerate [52, 53] and thus release their iron content into the extracellular space, which initiates an additional wave of oxidative stress [54, 55].

Clinically, the duration of the relapsing-remitting phase can vary between individuals [3]; however, the coerciveness of the progressive phase and the rate of neurological decline are highly consistent irrespective of the preceding disease course or its severity. This led to the assumption that neurodegenerative mechanisms in patients with progressive MS depend on the patients' age [4, 56]. Iron accumulates in the human brain in an age-dependent manner and iron-mediated amplification of oxidative stress may contribute to the age-related pathology of progressive MS [1, 57]. Iron accumulation is scarce in rodent models of multiple sclerosis and barely reflects the extent of oxidative injury observed in patients with multiple sclerosis [37].

**Figure 1:**
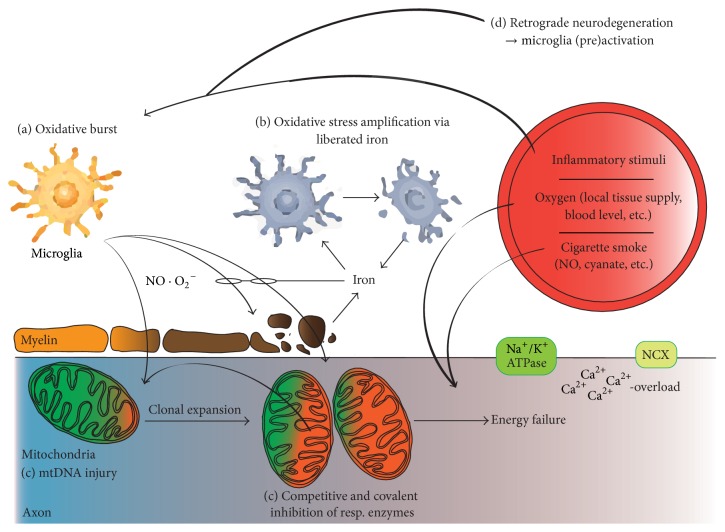
Oxidative stress-related mechanisms of tissue injury in multiple sclerosis. The temporal sequence and interconnection of cytotoxic events in MS may be different in individual lesions, stages of the disease, and different patients. Therefore, Figure 1 does not describe a timeline from events depicted in (a) to that in (d). (a) Microglia are activated by an unknown trigger pathology, the breakdown of the blood-brain barrier, and local and systemic inflammatory stimuli. Microglia activation itself may further impair the blood-brain barrier permeability. Microglia release nitric monoxide and superoxide molecules into the extracellular space. Nitric monoxide is uncharged and therefore penetrates lipid layers. Contrary to nitric monoxide, superoxide is unable to diffuse across biological membranes. It is rapidly converted into hydrogen peroxide, which, in contrast to superoxide, is able to diffuse into the extracellular environment. Additional amplification mechanisms involve microglia preactivation via axonal degeneration (d). (b) Iron is physiologically stored within the myelin sheets and liberated into the extracellular space upon demyelination. Extracellular iron amplifies oxidative stress as it travels between the ferrous and ferric states, inducing the production of highly reactive hydroxyl radicals. Iron is absorbed by microglia, which show histological signs of cell death under the high iron load and thus may release iron and initiate a second wave of oxidative stress. (c) Mitochondrial DNA (mtDNA) is vulnerable to free radical-mediated damage resulting in mtDNA deletions, which are found in neurons and axons of patients with multiple sclerosis. Mitochondria carrying such mutations are amplified by the clonal expansion in neurons. The mitochondrial respiratory chain is inhibited by covalent modifications caused by free radicals both competitively and irreversibly. A combination of these factors leads to energy failure via decreased ATP production. An important source of free radicals is the mitochondrial respiratory chain itself, particularly at low oxygen tension and reperfusion and in demyelinated axons. The exogenous factors, such as free radicals and cyanate delivered by smoking, inhibit mitochondrial function and cause demyelination in experimental conditions. Energy deficiency lowers Na^+^/K^+^-ATPase activity, resulting in the reverse operation of the Na^+^/Ca^2+^ exchanger (NCX) and thus increases Ca^2+^ levels. This event further activates the neurodegenerative and cell death pathways.

In the human brain, the highest iron content is measured in the deep grey matter nuclei, particularly in the basal ganglia [50, 52, 58]. However, lesion incidence and destructiveness are similar in the deep grey matter nuclei and the white matter of patients with multiple sclerosis [46, 59]. Histologically, the deep grey matter of patients with multiple sclerosis reveals a diffuse neurodegeneration pattern [60], quantitatively related to the patients' motor dysfunction prior to death [46]. The deep grey matter lesions of patients with multiple sclerosis show high levels of oxidative stress in neurons and oligodendrocytes. The level of oxidative stress in these lesions is positively associated with local iron load from both a topographical and a quantitative point of view [46, 52]. Similar to white matter lesions, two waves of iron liberation (Figure 1) have been suggested [46]. Inducible nitric oxide synthase (iNOS) is not expressed in the human central nervous system in baseline conditions [61, 62]. It is remarkable that the level of iNOS is elevated in the deep grey matter nuclei compared to the cortex or white matter in both patients with multiple sclerosis and controls [46]. Nitric monoxide is a competitive inhibitor of the respiratory chain [63, 64]. In high concentrations, in hypoxic conditions, or in the presence of superoxide, nitric monoxide promotes excitotoxicity and apoptosis [65, 66]. Low iNOS expression is cytoprotective in normoxic conditions and in the absence of superoxide [67]. In iron laden microglia cells, iNOS expression is upregulated [68, 69]. These findings suggest that iNOS upregulation could be an adapting mechanism in response to iron accumulation in the central nervous system and particularly in the deep grey matter [70]. Nitric monoxide mediated inhibition of the respiratory enzymes may also explain the clinical and pathological observation of early atrophy of deep grey matter in patients with Alzheimer's disease [71, 72] and that the deep grey matter nuclei are selectively vulnerable to hypoxemia or energy failure, in conditions such as carbon monoxide poisoning [73] and mitochondriopathies [74].

## 5. Hypoxia and Energy Failure

### 5.1. Mitochondrial Injury

The role of the mitochondria in the pathogenesis of multiple sclerosis was suggested by the observation that patients with Leber's hereditary optic neuropathy, a disease caused by mitochondrial DNA mutations, have an increased risk of developing multiple sclerosis-like Harding's disease [75]. In patients with multiple sclerosis, mitochondrial dysfunction has been described extensively in the cortex and white matter [1, 76]. Hypoxia-like lesions (pattern III multiple sclerosis lesions) [77] display various mitochondrial respiratory chain defects in axons, oligodendrocytes, and astrocytes [78]. Mitochondrial gene expression of 26 nuclear-encoded subunits of the oxidative phosphorylation chain and activity of complexes I and III are decreased in cortical neurons. The distribution of these “respiratory deficient” neurons is unknown, but they are not restricted to the areas of myelin loss [79, 80], suggesting a diffuse process. Reactive oxygen and nitrogen species are produced by activated microglia and macrophages through oxidative burst that releases these molecules into the extracellular space [81]. Nitric monoxide can diffuse across membranes and competes with oxygen for the binding site of mitochondrial cytochrome c oxidase; thus, it decreases respiratory chain function [82]. Reactive oxygen and nitrogen species induce covalent modifications and thus mutations in the mitochondrial DNA, which is more vulnerable than nuclear DNA [74, 83]. Mitochondrial DNA defects are present in patients with multiple sclerosis [80, 84]. Mitochondrial DNA mutations induced by reactive species inhibit the efficiency of oxidative phosphorylation and further increase the production of reactive oxygen species, thus leading to a vicious circle [85]. In normal conditions, 1-2% of the electrons escape from mitochondrial oxidative phosphorylation [86] and react with molecular oxygen producing superoxides in the mitochondrial matrix, in the compartment that contains mitochondrial DNA [87]. Hypoxia increases the production of reactive oxygen intermediates by deregulating the mitochondrial electron transport chain [88, 89]. Similarly, mitochondrial superoxide production increases when adenosine triphosphate (ATP) production is decreased (e.g., at a high membrane potential and pH gradient, low levels of coenzyme Q, and high nicotinamide adenine dinucleotide (NAD)/NAD+ ratio) [90, 91]. These conditions are present in demyelinated axons with an impaired conduction of saltatory axon potential transmission [18].

The detoxification of reactive oxygen species reversed mitochondrial and axonal injury in experimental settings [23], where the nitration of mitochondrial proteins precedes tissue injury and is present in the intact axons of animals with EAE [92]. In patients with multiple sclerosis, the proportion of mutant to wild-type mitochondrial DNA copies (heteroplasmy) in metabolically active postmitotic neurons increases with disease progression and increases age, via the clonal expansion of defective mitochondrial DNA [93]. Damaged mitochondria are normally removed from the cells by autophagy, which involves the formation of double-membrane structures called autophagosomes that fuse with lysosomes to degrade their content [94]. The autophagosome formation is inhibited by mTOR-dependent signalling pathways [94]. mTOR signalling is inhibited when nutrients are scarce, growth factor-related signalling is reduced, and ATP concentrations are low. In these situations, mTOR signalling is suppressed and biogenesis of the autophagosomes increases [95]. The reason why mitochondria with mutated mitochondrial DNA undergo clonal expansion and whether this process is related to changes in mTOR signalling in multiple sclerosis remains unknown [1].

Mitochondrial dysfunction and consequently ATP deficiency in multiple sclerosis lesions lead to the failure of sodium removal from the axoplasm into the extracellular space during action potential conduction [56]. In this condition, accumulated sodium is replaced by calcium ions by the reverse operation of the sodium-calcium exchanger (NCX). Calcium subsequently activates calpains, which initiate the proteolytic degeneration of cytoskeletal proteins [16, 96, 97]. In addition, aberrantly expressed voltage-gated calcium channels and glutamate receptors have been described in multiple sclerosis lesions, and their presence might amplify calcium toxicity [98, 99]. The ionic imbalance may be amplified by alterations in individual sodium channel subunits in multiple sclerosis lesions [100, 101].

### 5.2. Real/Virtual Hypoxia

The central nervous system is highly dependent on the continuous blood flow and mitochondrial metabolism that produces ATP [102]. This dependence is well illustrated by the large number of neurological disorders due to genetic alterations in mitochondrial and nuclear genes encoding mitochondrial proteins [74] and CNS injury in conditions of hypoxia or hypoglycaemia [73]. The hypoxic features of multiple sclerosis lesions including the expression of HIF-1-alpha, other hypoxia-related proteins, and elevated concentrations of lactate within lesions have been reported [103–110]. In patients with multiple sclerosis, diverse factors related to local oxygen supply/demand contribute to the endpoint of neurodegeneration. Brain inflammation reduces the oxygen supply due to oedematous tissue swelling [111, 112] and increases oxygen consumption by the presence of inflammatory infiltrates [113] and by the formation of a diffusion barrier to oxygen. The neurological deficits in animals with EAE are correlated with spinal cord white and grey matter hypoxia quantitatively, temporally, and spatially [113]. Similar findings are suggested in humans with multiple sclerosis. Brownell and Hughes determined the location of 1594 macroscopically visible plaques and reported “the peculiarity that these are situated on the boundary zones between major cerebral arteries, which have penetrated in this periventricular region to their further point of supply” [114]. These border zones between the major cerebral arteries (the so-called watersheds) have a decreased oxygen tension, and magnetic resonance imaging of 1249 patients with multiple sclerosis revealed high levels of lesion load in these watershed zones [115], which is consistent with previous findings [116]. A meta-analysis of voxel-based morphometric studies in patients with multiple sclerosis revealed that the cortex located within watershed areas is more severely affected by atrophy than other cortical regions [117]. However, the relative contribution of axonal degeneration and subcortical lesion frequency on primary neurodegeneration compared to other factors such as meningeal inflammation and cortical lesion formation in these cortical areas remains unknown.

Smoking can trigger multiple sclerosis, propagate disease progression, and transiently worsen the motor functions in patients with multiple sclerosis [118–120]. Cigarette smoke contains over 4500 potentially toxic components including reactive oxygen species, nitric monoxide, and cyanate [121, 122]. Cyanate inhibits the mitochondrial respiratory chain [123] and causes demyelination [124–126]. The exogenous inhibition of mitochondrial function via hypoxia and increased blood levels of cyanate and free radicals due to cigarette smoke inhalation may explain this environmental risk factor in the pathogenesis of multiple sclerosis; however, further experiments are warranted for confirmation.

## 6. Clinical Consequences

Energy failure, due to mitochondrial injury and oxidative stress, is a key player in the pathogenesis of multiple sclerosis. Early clinical research argued over the “relief by flush” therapy [127, 128]. Drugs such as histamine and amyl nitrite, which increase perfusion and thus may counteract energy failure, provided only temporary beneficial effects [129]. Similarly, hyperbaric oxygen has highly significant transient effects in the treatment of patients with multiple sclerosis but failed to show any lasting results (e.g., 100% oxygen at 2 atmospheres for 90 min once daily, for a total of 20 exposures) [130, 131]. The failure of hyperbaric oxygen therapy in patients with multiple sclerosis may be related to oxidative stress, which can increase in such conditions, similar to reperfusion injury [132] and the temporal timing of oxygen delivery in oxygen-sensitive periods [113]. A novel approach targets histotoxic “virtual” hypoxia by counteracting mitochondrial injury [76]. These drugs may be beneficial for patients with progressive MS because they may be able to cross the relatively intact blood-brain barrier [133]. The potential targets for boosting mitochondrial functions are substances that would particularly enhance the operation of peroxisome proliferator-activated receptor gamma coactivator 1 alpha (PGC1-alpha) [134, 135]. PGC1-alpha is a transcriptional cofactor, which binds and activates nuclear transcription factors that are involved in mitochondrial function. In addition, PGC1-alpha expression is reduced in the cortical neurons of patients with multiple sclerosis [136]. Cyclophilin D and p66ShcA are both involved in the formation of mitochondrial permeability transition pores and subsequent cell death signalling. Cyclophilin D and p66ShcA inactivation significantly reduced axonal damage in EAE [137, 138]. The gene delivery of superoxide dismutase 2, a mitochondrial scavenger of superoxides, ameliorates the axonal pathology in EAE [139]. Similar protective properties were reported for MitoQ, an antioxidant accumulating in the mitochondria [113, 140]. The anti-inflammatory drugs may penetrate the relatively intact blood-brain barrier and inhibit proinflammatory mediators that are released by T- and B-lymphocytes or microglia [1, 5, 8].

A conclusion, which genetic, experimental, and pathological investigations clearly suggest, is that there is no evidence for a single cause and thus therapeutic target of multiple sclerosis. Instead, multiple amplification steps orchestrate the clinically observed phenotype in susceptible individuals [141], which is well reflected in clinical trials. Drugs that target general or various inflammatory pathways such as blood-brain barrier permeability and immune suppression/modulation or combine additional cytoprotective properties have proven highly effective in the treatment of patients with multiple sclerosis [142–147]. It is important to note that the pharmacodynamics of such drugs may be highly specific (e.g., natalizumab blocks the VLA alpha-4 subunit); however, they interfere with the biological pathways that have very broad/unspecific effects on the organism (e.g., natalizumab prevents leucocytes from entering the central nervous system). The treatment strategies interfering with more specific downstream pathways proved not only less effective but also potentially dangerous [148–151]. The testing of new treatment regimes, particularly in clinical trials, may therefore benefit from combined approaches targeting different cell death pathways. Such approaches may involve anti-inflammatory therapy [1], protection against oxidative stress [152], mitochondrial injury [76], and hypoxic energy failure [113].
